# The Alimentary Tract of African Bony-Tongue, *Heterotis niloticus* (Cuvier, 1829): Morphology Study

**DOI:** 10.3390/ani12121565

**Published:** 2022-06-17

**Authors:** Maria Cristina Guerrera, Marialuisa Aragona, Marilena Briglia, Caterina Porcino, Kamel Mhalhel, Marzio Cometa, Francesco Abbate, Giuseppe Montalbano, Rosaria Laurà, Maria Levanti, Germana Germanà, Giacomo Zaccone, Krystyna Zuwala, Michal Kuciel, Antonino Germanà

**Affiliations:** 1 Zebrafish Neuromorphology Lab, Department of Veterinary Sciences, University of Messina, 98168 Messina, Italy; mguerrera@unime.it (M.C.G.); mlaragona@unime.it (M.A.); marilena.briglia@unime.it (M.B.); catporcino@unime.it (C.P.); kamel.mhalhel@unime.it (K.M.); marzio.cometa@unime.it (M.C.); gmontalbano@unime.it (G.M.); laurar@unime.it (R.L.); mblevanti@unime.it (M.L.); pgermana@unime.it (G.G.); agermana@unime.it (A.G.); 2 Department of Veterinary Sciences, University of Messina, 98168 Messina, Italy; zacconegiacomo@gmail.com; 3 Department of Comparative Anatomy, Institute of Zoology and Biomedical Research, Faculty of Biology, Jagiellonian University in Krakow, 30387 Krakòw, Poland; krystyna.zuwala@uj.edu.pl; 4 Poison Information Centre, Department of Toxicology and Environmental Disease, Faculty of Medicine, Jagellonian University, Kopernika 15, 30501 Krakòw, Poland; michalkuciel@gmail.com

**Keywords:** morphology, alimentary tract, *Heterotis niloticus*

## Abstract

**Simple Summary:**

*Heterotis niloticus* is a primitive freshwater teleost. It is a candidate for aquaculture in Africa with a good conversion rate and is used for evolutionary studies for its anatomical similarities with reptiles and birds. *H. niloticus* is also an endangered species for several reasons, including overexploitation. The purpose of the present study was to investigate, by gross anatomy and light microscope analysis, the morphological structure of the digestive system of the African bony-tongue, from the oropharyngeal cavity to the rectum, including its associated glands. A peculiar feature of this species is the presence of both bone trabeculae and well-defined cartilaginous areas in the process of ossification, in the deeper layers of the tongue. The so-called “African bony- tongue” is due to these characteristics. On both sides of the tongue, two tubular structures covered by numerous taste buds, as well as mucous cells, were found. The presence of well-defined lymphoid tissue in both pyloric ceca and rectum is described for the first time. Further investigations could aim to optimize husbandry and feeding protocols permitting, also, to understand the evolutionary process.

**Abstract:**

A morphological study of the alimentary tract, from the oropharyngeal cavity to the rectum, including the attached glands, of African bony-tongue, *Heterotis niloticus* (Cuvier, 1829) was carried out by gross anatomy, and light microscope analysis. This study aimed to give a deeper knowledge of the alimentary tract morphological features of this species of commercial interest. *H. niloticus* is distinguished by individual morphological characteristics showing a digestive tract similar to that of reptiles and birds. Within the oropharyngeal cavity, two tubular structures with digitiform ends are arranged on both lateral sides of the triangular tongue. The oropharyngeal cavity connects the stomach by a short esophagus. This latter is adapted to mechanical trituration, and it is divided into a pars glandularis and a thick-walled pars muscularis. The gizzard flows into the anterior intestine and two blind pyloric appendages, which exhibit specific functions, including immune defense for the presence of secondary lymphoid organs. The anterior intestine continues with the middle and posterior tracts up into the rectum. According to the histological observations, all regions of the alimentary tract have common structural features, typical of hollow organs, with differences in the mucosa structure that reflects the different functions of the apparatus, from mouth to anus. Within this study, we provided the first basis for future studies on optimizing rearing conditions, feed conversion ratio, and the digestive capacity, improving the growth performance of this species, and ensuring its conservation.

## 1. Introduction

*Heterotis niloticus,* known as African bony-tongue, is a large primitive freshwater teleost with a highly compressed cylindrical body at the sides, inhabiting many rivers, streams, natural and artificial lakes of the Nilo-Sudanese region, Central and West Africa [1,2,3,4,5]. It has also been found in swamps and floodplains, which proves its capability to survive in deoxygenated waters [3,6,7]. It belongs to the family Arapaimidae and represents the only species of the genus *Heterotis* [8]. Its basal position in the general fish phylogeny as Osteoglossiforms put it in an important taxon of interest for evolutionary processes studies [9]. This species is of great interest also for other reasons. First, its great growth performance makes it a leading candidate species with high aquaculture potential. Finally, Dudgeon [10] emphasized the risk of extinction for this species due to fishing pressure and the need to ensure its conservation with appropriate measures and management strategies [11]. African bony-tongue can be considered an opportunistic omnivore according to the diversity in the composition of its diet as well as in its seasonal variation [2]. Its diet is mainly based on fish, mollusks, and crustaceans but also aquatic invertebrates, terrestrial insects, and seeds [1,2,11]. Among fish belonging to Osteoglossids, the African bony-tongue is the only one that feeds extensively on plankton. Bake and Sadiku [12] define African bony-tongue as predominantly planktivorous, while other authors support that African bony-tongue is an omnivore rather than a detritivore, a planktivory or a specialist invertebrate feeder [2,13]. Other authors refer to African bony-tongue as a carnivore rather than a piscivore [2,14,15,16]. The anatomy of the gastrointestinal tract of fishes, from the oropharyngeal cavity to the rectum, shows different morphological and functional variations which reflect the phylogeny, ontogeny, diet, and environment [17]. The function of the digestive tract is complex. It is involved in ingestion, secretion, digestion, absorption and the elimination of waste substances [18]. The oropharyngeal cavity of vertebrates plays an important role in the collection and initial processing of food [19,20]. Adaptive morphological changes are present in the oropharyngeal cavity of different fishes strictly related to the feeding habits of the fish [21,22,23,24,25]. The tongue, which in most fish species is defined as a thickening of the floor of the mouth [26,27], in some species is described as a real taste organ due to the presence of taste buds scattered on its dorsal surface, demonstrating a gustatory ability [28,29,30,31,32]. In addition, the presence of numerous canine-like teeth on the dorsal surface gives the tongue a determining role in the capture of live preys [29,32]. Immediately behind the mouth and parallel to branchial arcs, we find the pharynx in continuity with the esophagus. In carnivorous fish, the esophagus has more mucous cells and a thinner muscular layer than in herbivorous fish, preventing congestion during the swallowing of prey [33,34]. The stomach of a carnivorous fish can generally be divided into two compartments, cardio fundic and pyloric, with the identification of cardiac, pyloric, and fundic regions [35]. Stomachless fishes account for approximately 20% of species, including many Cyprinids and Cyprinidonts [36], while a gizzard is only found in a relatively few species, among which African bony-tongue [5,37]. Pyloric caeca are not present in all fish species, and even species having it show an extreme intraspecies variations. Their function is poorly understood, and the secretion of active enzymes in the gut and the neutralization of the chyme acidity were hypothesized. They may play more or less complex roles in the digestive cycle in different fish species. In African bony-tongue, Agbugui et al. [37] attribute to the pyloric ceca a role in storing, absorbing and digesting, mostly of carbohydrates. Whereas the caeca of mammals and birds are fermentation chambers, fish caeca are an adaptation to increase gut surface area [38]. Bowel length in herbivorous fish is greater than that of omnivores, which in its turn, is longer than those of carnivores. Those findings can be explained by the nature of the diet, in particular, the number of vegetal materials in diets [18,38]. Numerous comparative studies with other vertebrate species such as birds and reptiles are present in the literature [20,22,23,32,39,40,41,42,43,44]. Although few data concerning the digestive tract of African bony-tongue are present in the literature [45,46], they appear incomplete with minimal descriptions of both gross anatomy and histological features. For this reason, we investigated in depth the morphological structure of the various segments of the digestive system of African bony-tongue, from the oropharyngeal cavity to the rectum, including its associated organs (liver and pancreas) for the first time. We aim with this study to give a deeper knowledge of the alimentary tract morphological features of the African bony-tongue, a species of commercial interest, which may provide the first basis for futures studies on optimizing rearing condition, feeding protocols, food conversion and the digestive capacity of adult African bony-tongue, improving the growth performance of this species and ensure its conservation.

## 2. Materials and Methods

In this study, n° six pooled samples of males and females adult African bony-tongue *Heterotis niloticus* (Cuvier, 1829) with a mean total length (TL) of 27 cm (±0.94), and a standard length (SL) of 25.5 cm (±0.77) were used. The specimens were collected in the Congo River at Kingabwa and the lake Mai-Ndombe (Congo). After the fish were caught, they went to the local exporter’s tanks where they were fed and quarantined for 7 days. Then, fish were transported to Poland where water and temperature acclimation took place in rearing tanks. Here, 24 °C, 15/09 light-dark cycle were maintained for 4 weeks. Fish were fed were feed twice a day with commercial pellets (Sera Stor Pellets Nature) and were starved for 12 h before being euthanized.

After they had been euthanized using 0.3% tricaine (MS-222, Syndel Laboratories Ltd., Qualicum Beach, BC, Canada), each specimen was incised to expose the digestive tract, to favor the penetration of the fixative, 10% formaldehyde, inside the celomatic cavity. Finally, they were transported, in 70% ethanol, to the Sicilian laboratory in Italy to be processed. The tongue, as well as the digestive tract, and the liver have been sampled. The digestive system was divided into segments: esophagus, stomach, pyloric caeca, and intestine. The intestine was divided into anterior, middle, posterior tracts, and rectum considering the tridimensional anatomical disposition. We have defined the anterior intestine as the segment originating with pyloric ceca, in a craniocaudal direction, from the gizzard to the first fold of the digestive tract. The middle intestine is the segment bordered by the first and the second folds in a caudocranial direction. The posterior intestine, however, was defined as the tract starting from the second fold of the intestine until the 45° curved part of the intestine which represents the rectum, ending up in the anus. The organs were processed for routine histological study (dehydration, diaphanization, and paraffin embedding (for the technique in detail see [22,23]). The histological investigation was carried out on the esophagus, stomach, intestine and pyloric caeca, which in turn were divided into three, two (proventriculus and ventriculus), four (anterior, intermediate, posterior, and rectum) and eight segments respectively. 

### 2.1. Histochemical Staining

The different intestinal segments were cut and slice sections were obtained, rehydrated and stained with Masson’s trichrome with aniline blue containing four different dyes: Weigert’s iron hematoxylin for nuclei, picric acid for erythrocytes, a mixture of acid dyes (acid fuchsin—“ponceau de xylidine”) for cytoplasm and aniline blue for connective tissue. The stained sections were observed under an OLYMPUS BX51 system Microscope (Olympus optical Co., Ltd., Nagano, Japan), and micrographs were taken using a digital camera OLYMPUS DP12 (Olympus optical Co., Ltd., Nagano, Japan). 

### 2.2. Immunocytochemical Staining

For laser confocal microscopy 3 tongues were processed for routine paraffin embedding. The blocks were cut in 10 μm thick serial sagittal or horizontal sections, mounted on gelatin-coated microscope slides and processed for immunofluorescence. Some serial sections were washed with Tris-HCL (0.05 M, pH 7.5) containing 0.1% bovine serum albumin and 0.2% Triton X-100. The endogenous peroxidase activity and non-specific binding were blocked (3% hydrogen peroxide and 50% fetal bovine serum), and the sections were incubated in a humid chamber overnight at 4 °C with anti-vimentin (Serotec, 201100, dil. 1:100). Subsequently, the sections were rinsed in buffer and incubated for 40 min at room temperature with Alexa fluor 488 donkey anti-mouse IgG (H + L) (Invitrogen, A 21202 dil. 1:300) in dark humid chamber. Finally, the sections were washed, dehydrated, and mounted with Fluoromount Aqueous Mounting Medium (Sigma Aldrich, Burlington, MA, USA). Sections were analyzed, and images were acquired using a Zeiss LSMDUO confocal laser scanning microscope with META module (Carl Zeiss MicroImaging GmbH, Munich, Germany) microscope LSM700 AxioObserver. Representative sections were incubated with non-immune rabbit sera instead of the primary antibodies, or omitting the primary antibodies, following the same procedure described above providing negative controls. Under these conditions, no positive immunostaining was observed (data not shown). 

All the procedures for animal care, handling and tissue removal were conducted with the ethical principles indicated by the European Union Directive (63/2010EU) on the use of animals for scientific purposes.

## 3. Results 

**Figure 1:**
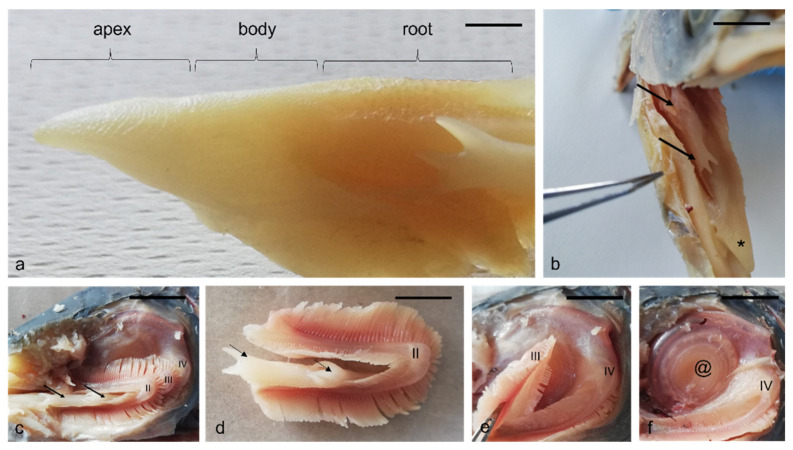
Gross anatomy. (**a**) The macroscopical aspect of *H. niloticus* tongue with three different areas: an apex, a body, and a root. (**b**) The opened oropharyngeal cavity showing the apex (asterisk), tubular organs on the sides of the tongue with digitiform ends (arrows). (**c**,**d**) Tubular organs (arrows) associated with the second gill arch (II). The third gill arch (Ⅲ) (**e**,**f**) The fourth branchial (IV) arch modified in a spiral-shaped filtering apparatus (@). Scale bar: 1 cm.

The digestive apparatus of the African bony-tongue showed interesting morphological features. The tongue has an isosceles triangle shape with a pointed apex (Figure 1a). In the tongue of the African bony-tongue a free apex, a body and a root are shown (Figure 1a). There are two tubular organs, on both sides of the tongue, with digitiform ends, associated with the second gill arch, projecting into the oropharyngeal cavity (Figure 1b–d). The fourth branchial arch appears modified in a spiral filtering apparatus, very similar in the morphology and gross anatomy to the human cochlea (Figure 1e,f).

**Figure 2:**
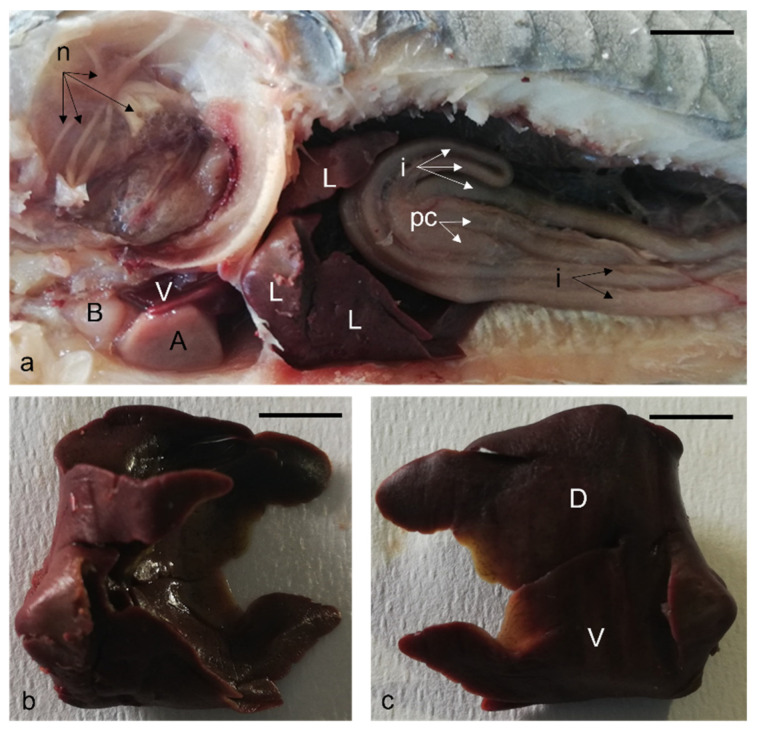
Gross anatomy. (**a**) Photographs of the left side of the celomatic cavity of *H. niloticus*; In cranial position: left lobe of the liver (L); pyloric appendages (pc); intestine (i); bulbous (B), atrium (A), ventriculus (V) of the heart; in dorsal position: visible innervation (n) after spiral-shaped apparatus removal. (**b**) Photographs of the liver left side. (**c**) Liver right side: dorsal (D) and ventral (V) lobes. Scale bar: 1 cm.

After spiral filtering apparatus removal, a rich innervation was visible (Figure 2a). The digestive tract occupies the entire celomatic cavity (Figure 2a). The large liver fills most of the cranial portion in the celomatic cavity. It also wraps entirely around the stomach and shapes itself in the surrounding space (Figure 2a). Furthermore, it is rotated to the right according to a median longitudinal axis. The liver, isolated from the celomatic cavity, is bilobed with two faces, the left (Figure 2b) and the right sides (Figure 2c). Therefore, the right lobe and the left lobe are respectively dorsal and ventral (Figure 2c) and completely envelop the gizzard right face. 

**Figure 3:**
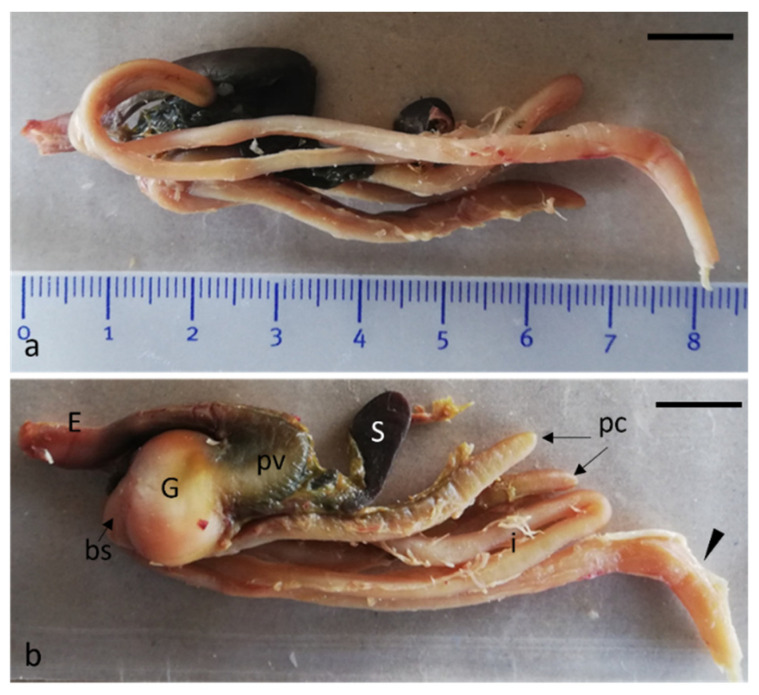
Gross anatomy. (**a**) Photograph of the left side of the gastrointestinal tract. (**b**) Photograph of the right side of the gastrointestinal tract: esophagus (E), proventriculus (pv), gizzard (G), cranioventral blind sac (bs), spleen (S), pyloric appendages (pc), intestine (i), rectum (arrowhead). Scale bar: 1 cm.

The digestive system has two different views depending on the observation side: from lateral left (Figure 3a) and right (Figure 3b) view. It is characterized by an intestine curling around to the left side of the esophagus and bending to form numerous loops ending at the rectum. The rectum is the last part of the intestine, ending in the anus. It is possible to see, thanks to the transparency of the bowel wall, the colon-rectal valve, a muscle sphincter, and the posterior intestine, which is flexed ventrally by 45° (Figure 3a,b). 

**Figure 4:**
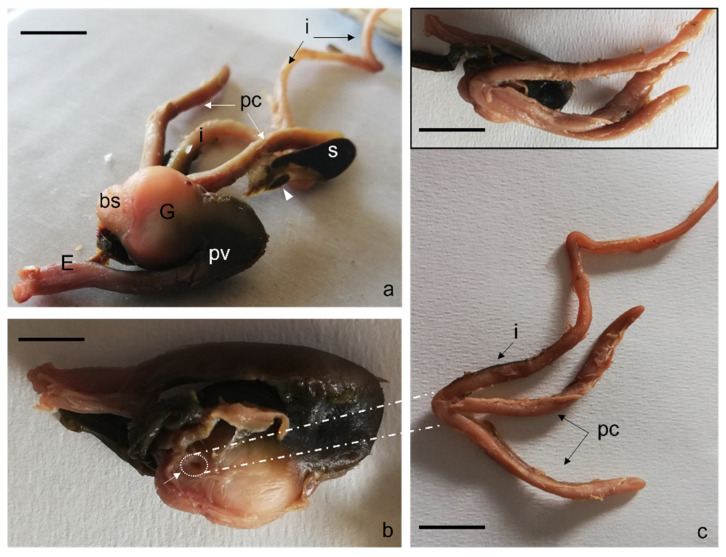
Gross anatomy. (**a**) Photograph of the right side of the gastrointestinal tract: esophagus (E), proventriculus (pv), gizzard (G), cranioventral blind sac (bs), spleen (s), accessory or supernumerary spleen (arrowhead), pyloric appendages (pc), intestine (i). (**b**) Photographs of the right side of gizzard: pyloric ostium (arrow) visible after intestine and pyloric appendages removal. (**c**) Isolated intestine (i) and pyloric appendages (pc), anatomical relationships of the intestine and the pyloric appendages with the gizzard (insert). Scale bar: 1 cm.

Following a consequential anatomical order, the esophagus, proventriculus or glandular part of the stomach, gizzard or pars muscular with blind sac, the intestine, and the two pyloric caeca, were analyzed. Accessory or supernumerary spleens were identified in African bony-tongue (Figure 4a). Two pyloric ceca are placed after the pyloric sphincter, where the stomach junction with the intestine is located (Figure 4b,c). More specifically, the two blind pyloric appendages, originate as a three-way crossroads/intersection together with the duodenum, from the cranial parts of the right surface of the gizzard through a pyloric opening (Figure 4b). The feed is conveyed into the ventral skull blind sac and then through the pyloric orifice into the three-way intersection with the two pyloric caeca in a ventral position and the anterior intestine in a dorsal position (insert Figure 4c). 

### Histological Features

**Figure 5:**
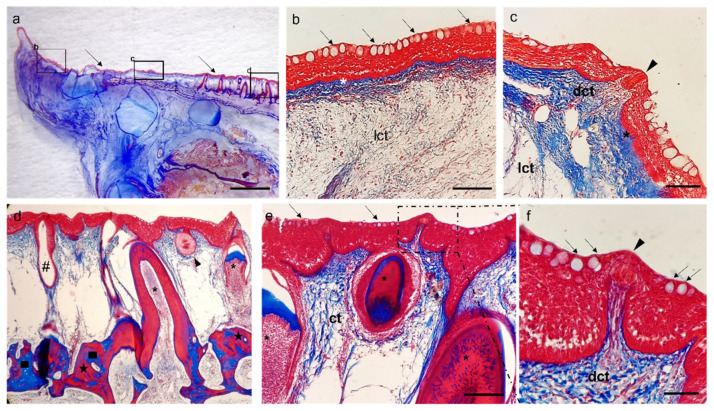
Masson Trichrome with Aniline Blue staining. (**a**) Stereomicrograph of a sagittal section of the tongue: tongue dorsal surface (arrows) covered by several papillae. (**b**) Light micrograph of tongue apex, corresponding to insert b: stratified epithelium with abundant caliciform cells (arrows), basal membrane (asterisk) connecting the epithelium and loose connective tissue (lct). (**c**) Mucosa of the tongue body raising in folds and showing taste buds (arrowhead), basal membrane (asterisk), dense connective tissue (dct) projecting at taste bud base; loose connective tissue (lct) in deeper layers. (**d**) Light micrograph of tongue root: villiform teeth in various eruption stages (asterisks), glandular extraepithelial and intramural bodies (arrowhead), excretory ducts (#), compact bone tissue (stars) replacing the pre-existing cartilaginous tissue (squares). (**e**) Scattered taste bud (dotted box), connective tissue (ct), teeth (asterisks), goblet cells (arrows). (**f**) Taste bud (arrowhead), dense connective tissue (dct), goblet cells (arrows). Scale bar: 500 μm (**a**), 200 μm (**d**), 100 μm (**b**,**e**), 50 μm (**c**,**f**).

The free apex of the tongue is covered by stratified epithelium with abundant caliciform cells inserted between the lining cells (Figure 5b). At the lingual dorsal surface, the mucosa is raised in folds and shows taste buds at both body (Figure 5c) and root levels (Figure 5d). In addition, villiform teeth in various eruption stages, glandular extraepithelial and intramural bodies as well as related excretory ducts and scattered taste buds, were observed at the root level (Figure 5c–f). Under the epithelium, there is a connective tissue that appears dense due to its high prevalence of the fibrillar component (Figure 5b–d,f,g) and its rich vascularization, close to the epithelium (Figure 5c). However, in the underlying layers, the dense connective tissue gives way to the loose connective with a less dense fibrillar component consisting of thin and scattered fibers and a prevalent amorphous component (Figure 5a,b,e,f). 

**Figure 6:**
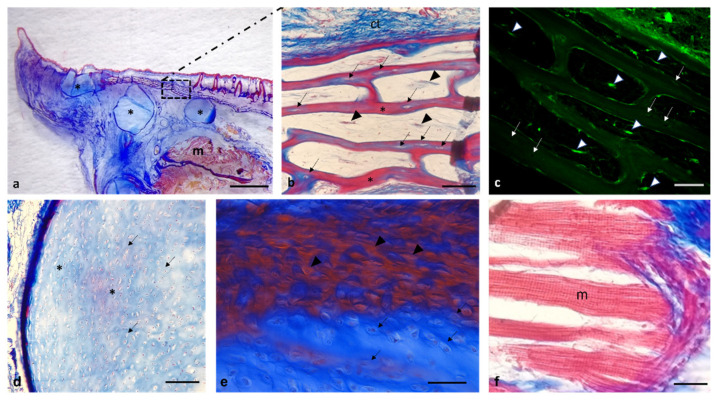
Masson Trichrome with Aniline Blue staining. (**a**) Stereomicrographs of tongue sagittal section: cartilaginous nuclei (asterisks), striated muscle fibers (m), bone trabeculae (dashed rectangle). (**b**) Light micrograph of tongue sagittal section: walled osteocytes (arrows) in the calcified matrix (asterisks), star or fused-shaped mesenchymal cells (arrowheads) in mesenchymal tissue areas delimited by anastomosed bone trabeculae. (**c**) Laser confocal micrograph: vimentin positive cells (arrowheads), osteocytes (arrows). (**d**) Chondrocytes (arrows) immersed in an abundant extracellular matrix (asterisks). (**e**) Ossification front: chondrocytes (arrows) and osteocytes (arrowheads) immersed in the bone matrix (**f**) Striated muscle fibers (m). Scale bar: 500 μm (**a**), 50 μm (**b**,**d**–**f**), 5 μm (**c**).

Limited to the body and root portions, the presence of bone trabeculae is noted justifying the definition of bony tongue (Figure 6a). Well-defined cartilaginous areas were found in the deeper layers of the tongue apex, body and root (Figure 6a).

At greater magnification, walled osteocytes are visible in the calcified matrix (Figure 6b). These anastomosed bone trabeculae delimit the containing mesenchymal tissue areas in which vimentin-positive cells were evident. (Figure 6c). In the cartilaginous areas described above chondrocytes are embedded within an abundant extracellular matrix (Figure 6d). Red stained osteocytes included in the bone matrix (Figure 6e) and striated muscle fibers (Figure 6f) were shown.

**Figure 7:**
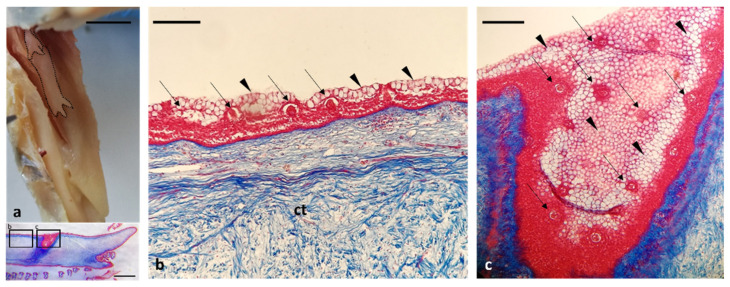
(**a**) Gross anatomy: photographs of the tubular organs with digitiform ends (dotted area), stereomicrographs sagittal sections of digitiform ends (insert). (**b**) Light micrograph corresponding to the b insert: mucosa with several taste buds (arrows) and mucous cells (arrowheads). (**c**) Light micrograph corresponding to the c insert: invaginations or pockets-like structure richly covered by taste buds (arrows) and mucous cells (arrowhead). Masson Trichrome with Aniline Blue staining (**a**–**c**). Scale bar: 1 cm (**a**), 500 μm (insert, in the box below (**a**)), 100 μm (**b**), 50 μm (**c**).

The two tubular structures, arranged on both sides of the tongue (Figure 7a), were characterized by a layered floor epithelium (Figure 7b). This latter is covered by numerous taste buds as well as mucous cells (Figure 7b). In addition, it was registered the presence of invaginations richly covered by taste buds (Figure 7c). 

African bony-tongue gastrointestinal tract wall is made up of different layers: mucosa, submucosa, muscolaris externa and adventitia or serosa.

**Figure 8:**
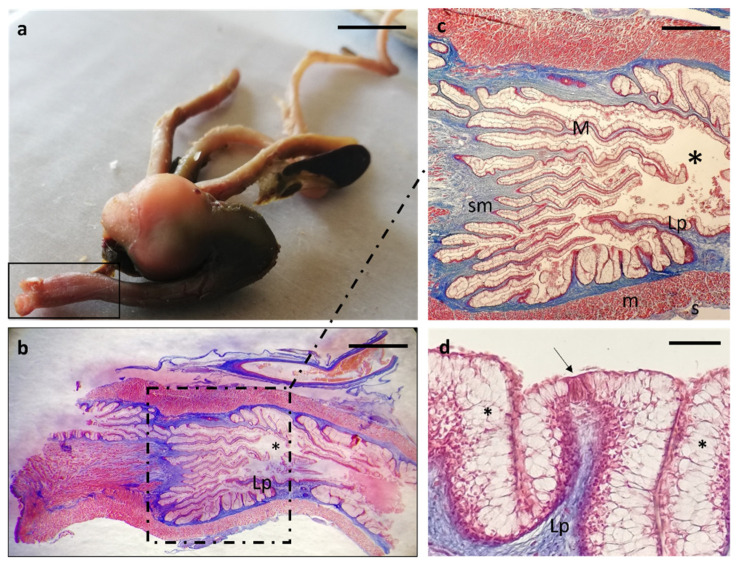
(**a**) Gross anatomy of the gastrointestinal tract: esophagus (rectangle). (**b**–**d**) Masson Trichrome with Aniline Blue staining. (**b**) Stereomicrographs of the esophagus sagittal section: goblet cells (asterisk), lamina propria (Lp). (**c**) Histological organization of the esophagus of *H. niloticus*: sagittal section showing mucosa (M), lamina propria (Lp), submucosa (sm), internal muscular layer (m), serosa (S). (**d**) Micrograph of a mucosa detail: taste bud (arrow); goblet cells (asterisks) lamina propria (Lp). Scale bar: 1 cm (**a**), 500 μm (**b**), 100 μm (**c**,**d**).

The disposition of the major muscle component remained relatively constant, while the mucosa showed structural differences in the various regions of the apparatus. The esophagus appeared as a muscular tube that leads to the stomach (Figure 8a). The mucosa and submucosa tunicae of the esophagus were raised in folds protruding into the lumen (Figure 8b,c). The mucosa was covered by a stratified epithelium characterized by a high density of mucous secreting cells. Sporadic taste buds were present embedded in esophagus stratified epithelium as in other teleosts (Figure 8b). The submucosa was made up of dense connective tissue rich in elastic and collagen fibers. The presence of the esophageal pharyngeal sphincter was evident. The muscular tunica was characterized by striated muscle fibers. It was thick with outer and inner circular longitudinal layers. Moving caudally, the folds of the mucosa become higher, with a villiform aspect and the muscular tunica was thinner and represented only by the external circular layer (Figure 8a–c).

**Figure 9:**
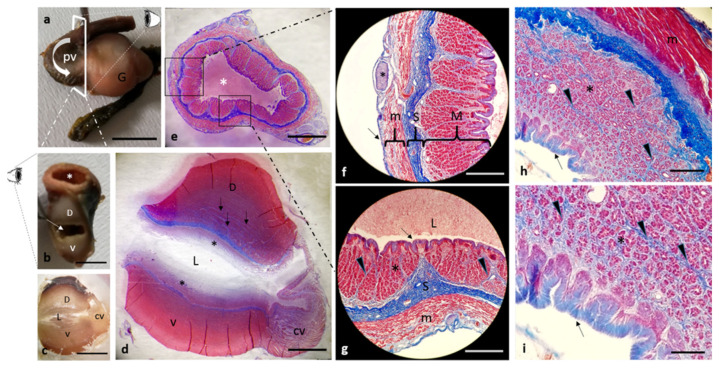
(**a**) Stereomicrograph: proventriculus (pars glandularis) (pv), gizzard or ventricle or pyloric stomach (pars muscularis or ventriculus) (G), transversal section plane through proventriculus (rectangle). (**b**) proventriculus small cranial part (asterisk), isthmus connecting the wider caudal part of proventriculus with ventriculus (arrow), dorsal (D) and ventral (V) ventricle muscles. (**c**) Dorsoventral/sagittal section through thick muscles of the gizzard: dorsal muscle (D), ventral muscle (V), lumen (L), cranioventral blind sac (cv). (**d**–**i**) Masson Trichrome with Aniline Blue staining. (**d**) Stereomicrographs of a sagittal section of the gizzard showing biconvex lens-shape: dorsal muscle (D), ventral muscle (V), lumen (L), cranioventral blind sac (cv), gastric cuticle (asterisks), connective tissue septa infiltrating the bundles of muscle fibers (arrows). (**e**) Stereomicrograph of a transversal section of proventriculus: lumen (asterisk). (**f**) Transversal sections of proventriculus: mucosa (M) submucosa (S), muscularis (m), serosa (arrow), myenteric system (asterisk). (**g**) Transversal section of proventriculus: lumen (L); epithelium (arrow); serous cardic glands (asterisk), submucosa (S), muscularis (m), Connective tissue septa (arrowheads). (**h**,**i**) High magnification of a sagittal section through the wider caudal part of proventriculus: glands (asterisk), epithelium (arrow), muscles (m). Scale bar: Scale bar: 1 cm (**a**–**c**), 500 μm (**e**,**d**), 100 μm (**f**,**g**), 50 μm (**h**,**i**).

The serous tunica represents the outermost tunica of the esophagus portion. Without clear limits, the esophagus extends to the stomach. The latter consists of two distinct parts: the first is proventriculus (pars glandularis) which was characterized by a small cranial portion consisting of a small cranial part and a wider caudal part; the second was a biconvex lens-shaped ventricle or pyloric stomach called gizzard (pars muscularis or ventriculus) (Figure 9a).

**Figure 10:**
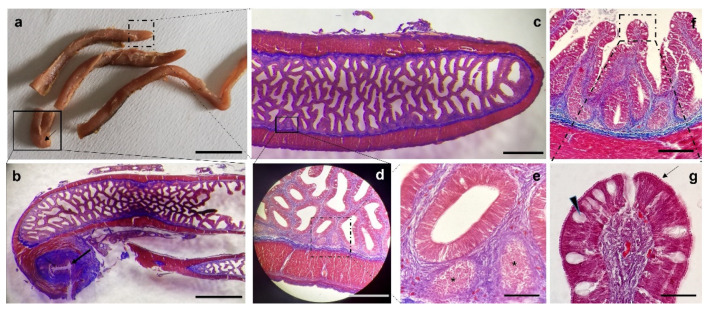
(**a**) Gross anatomy of the intestine and pyloric ceca. (**b**–**g**) Masson Trichrome with Aniline Blue staining. (**b**) Stereomicrographs of a sagittal section of pyloric ceca: *pyloric ostium* (arrow). (**c**) Stereomicrograph of a longitudinal section of the pyloric cecum with a labyrinthine aspect. (**d**,**e**) longitudinal section of the pyloric cecum: masses of lymphoid tissue in the mucosal lamina propria (dotted area and asterisks). (**f**) Light micrograph of the pyloric cecum in transversal section. (**g**) transversal section of pyloric cecum: microvilli (arrow) and goblet cells (arrowhead). Scale bar: 1 cm (**a**), 1 mm (**b**), 500 μm (**c**), 100 μm (**d**), 50 μm (**e**–**g**).

In a transverse section conducted through the full thickness of the proventriculus, it was possible to see the lumen of the continuous tract to the esophagus and the opening towards the ventricle or gizzard through the isthmus (Figure 9b). Around the isthmus, the dorsal and ventral muscles of the gizzard were seen in section (Figure 9b–d). In its initial stretch before the assumption of the curved structure, the proventriculus showed an oval section with a typical histological characteristic of hollow organs consisting of four main tunicae (Figure 9e). The mucosa at high magnification showed serous cardiac glands and was covered by a simple columnar epithelium. The submucosa was composed of dense connective tissue that sends both trophic and mechanical supporting septa to the glandular bodies of the overlying mucosa. The muscular layer consisted of smooth muscle fibers with circular orientation. A thin layer of longitudinal muscle fibers was surrounded by the outer tunica, the connective serosa. The myenteric nervous system between the serosa and circular layers of muscularis externa was also visible (Figure 9f,g). The stomach appeared as a dilated tract of the gastrointestinal apparatus which receives food from the esophagus. The mucosa of the gizzard was covered by a gastric cuticle except for the blind sac. Connective tissue septa infiltrated the bundles of muscle fibers, forming a three-dimensional network. Once digested, the feed bolus is deposited in the skull-ventral blind sac, and under longitudinal, transverse, and circular muscle fibers contraction is pushed to end up in the pyloric ostium, the pyloric caeca, and the duodenum (Figure 10a,b).

The general structure of the pyloric ceca was similar to the rest of the anterior intestine with a raised mucosa in folds covered by a simple columnar epithelium with microvilli and numerous intercalated goblet cells (Figure 10c,f,g). The mucosa was raised in villus-like folds anastomosed to each other in the proximal third, where debris was found near the microvilli. Moreover, the evident labyrinthic structure in both longitudinal and transversal sections of the pyloric appendix was shown (Figure 10c,d,f). The most typical feature of pyloric ceca was the presence of masses of lymphoid tissue in the mucosal lamina propria (Figure 10d,e).

**Figure 11:**
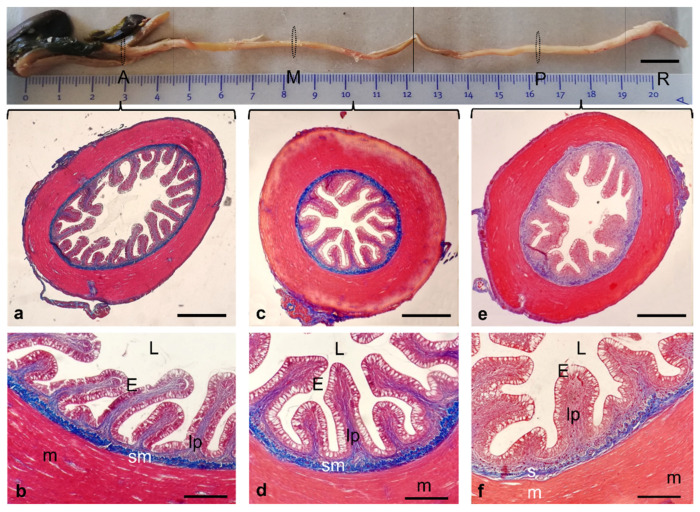
Gross anatomy: photographical insert of the gastrointestinal tract of *H. niloticus*; intestinal segments: anterior (A), intermediate (M), posterior (P), and rectum (R). (**a**–**f**) Masson Trichrome with Aniline Blue staining. (**a**,**b**) Micrographs of transversal sections of anterior, (**c**,**d**) intermediate and (**e**,**f**) posterior intestine. (**b**,**d**,**f**) Micrograph of the intestinal mucosa: epithelium (E), lumen (L), lamina propria (lp), submucosa (sm), muscularis (m). Scale bar: 1 cm (insert), 100 μm (**a**–**c**), 50 μm (**d**–**f**).

The intestine consisted of four segments: anterior, intermediate, posterior, and rectum (Figure 11). The anterior intestine (Figure 11a,b) comprised the simple columnar epithelium with prominent longitudinal folds and intercalated goblets cells, which secrete mucins to form mucus. The lamina propria was a loose connective tissue ensuring supporting and trophic functions for the lining epithelium. The mucosa was raised in longitudinal folds. The submucosa consisted of a dense network of connective tissue and blood vessels with a rich fibrillar component. The muscular tunica consisted mainly of a very thick inner circular layer and a very thin layer of fibers with an external longitudinal orientation. A thin serosa richly vascularized surrounded the perimeter of the organ. The intermediate (Figure 11c,d) and posterior intestinal mucosa (Figure 11e,f) showed variations related to functions. At the serosa level, a portion of the pancreas was visible (Figure 11c).

**Figure 12:**
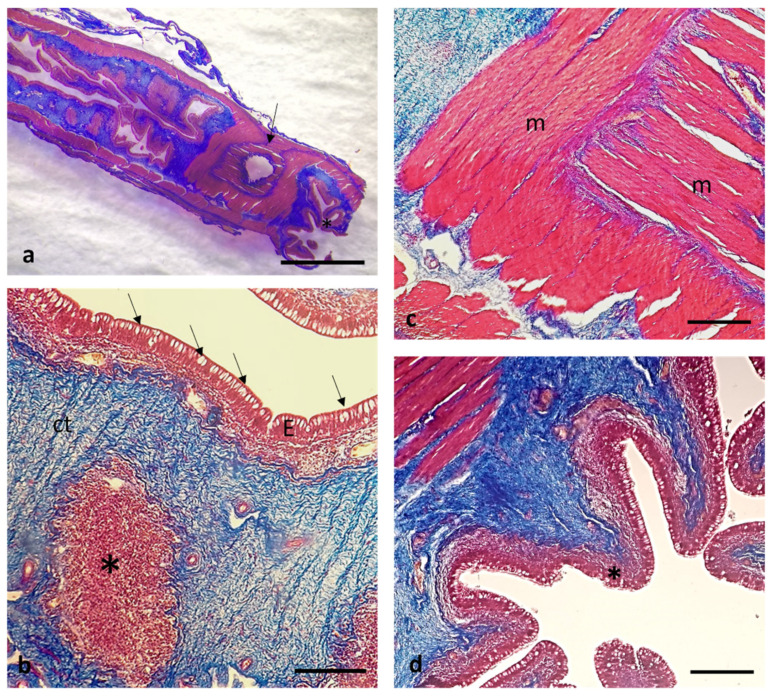
Masson Trichrome with Aniline Blue staining. (**a**) Stereomicrographs of the longitudinal section of the rectum: anal sphincter (arrow), anus (asterisk). (**b**) Micrograph of rectal mucosa: simple columnar epithelium (E), goblet cells (arrows), secondary lymphoid organs (asterisk) in the submucosa. (**c**) Muscular fibers (m) oriented in intertwined bundles forming an anal sphincter. (**d**) Micrograph of the anus (asterisk). Scale bar: 1 mm (**a**), 100 μm (**b**), 50 μm (**c**,**d**).

The rectum mucosa (Figure 12a) appeared covered by a simple columnar epithelium with abundant goblet cells compared to the rest of the intestinal segments. In the thickness of the submucosa also at the level of the rectum, secondary lymphoid organs were found (Figure 12b). Different oriented muscular fibers were grouped in intertwined bundles to form an anal sphincter located at the end of the rectum (Figure 12c) that flowed into the anus opening outwards (Figure 12d).

**Figure 13:**
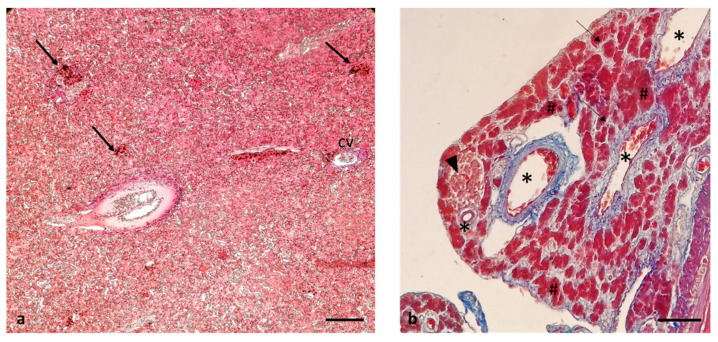
Masson Trichrome with Aniline Blue staining. (**a**) Micrograph of the liver: scattered melano-macrophage centers also known as macrophage aggregates (arrows), central lobular vein (cv). (**b**) micrographs of the pancreas: connective septa (arrows), acini formed by secretory cells (#), insular endocrine component (arrowhead) near the vessels (asterisks). Scale bar: 5 μm (**a**), 50 μm (**b**).

The liver of African bony-tongue showed no evident interlobular septa for which it lacks a lobulation. The organization of the parenchyma was characterized by very compact hepatocyte chains. There were scattered melano-macrophage centers also known as macrophage aggregates (Figure 13a). The pancreas was disseminated. The pancreas was associated with the intestinal wall: it was possible to observe, in association with sections of the intestine, the organization of the pancreatic acini and the ductal system, with the excretory ducts. Connective septa were visible. At higher magnification, it was possible to see that the acini were formed by secretory cells that project towards the lumen of a small duct. Also, an insular endocrine component was visible near the vessels (Figure 13b).

## 4. Discussion

Aquaculture contributes significantly to food supply around the world, especially in developing countries, such as in sub-Saharan Africa, where population growth is constant and fishery products cannot meet the demand for fish [47]. The knowledge of the morphological characteristics of the digestive system of fish is important to understand their alimentary physiology and optimize the production yield, improving the feed conversion and preventing disease [36,48]. Knowledge of nutritional physiology and digestive tract of fish can directly affect FCR (feed conversion ratio) and growth and, as a result, aquaculture production. In central-western Africa, the African bony-tongue represents an important food resource and, consequently, a source of income. However, although some studies on the digestive system of African bony-tongue have been conducted [32], literature is still scarce. We would like to enrich the currently available scientific knowledge with more precise details correlating gross and microscopic anatomy. Therefore, the purpose of this manuscript was to provide a detailed description of the digestive tract and its associated organs of the African bony-tongue, an emerging candidate fish species for the expansion of aquaculture in developing countries. Its use for intensive aquaculture requires further and more detailed studies to optimize yield and consider environmental sustainability. Starting from the oropharyngeal cavity, we observed a free apex triangular tongue with two tubular organs. The latter are associated with the second branchial arch, while the fourth branchial arch is converted into a spiral filtering apparatus. In many species of filter-feeding fishes, the presence of aligned organs to the branchial arches has been described [49,50]. These organs were identified as epibranchial organs with the role of taste perception and mediation in the aggregation and ingestion of planktonic food [51]. Other authors have described a snail-shaped epibranchial organ associated with the fourth branchial arch [50]. In literature, the mucus secretion function, useful to trap phytoplankton and bits of organic matter to then swallow them, was attributed to the spiral filtering apparatus. Furthermore, some authors believe that this organ may have a role in gas exchanges, food intake, or sensory-related mechanisms [48]. Tubular organ involvement in sensory function or in balance regulation could be suggested by a rich innervation visible after filtering apparatus removal. The intestinal tract is rotated to the right according to a median longitudinal axis and it curls around to the left side of the esophagus as in the teleost genus Arapaima [52]. Its initial portions, the proventriculus, and the gizzard are shared with crocodiles [53]. As Scadeng et al., [54] found in *Arapaima gigas*, accessory or supernumerary spleens are identified in *H. niloticus*. Both in *A. gigas* [55] and *H. niloticus* two pyloric ceca mark the beginning of the gut. They have been founded in some fish species, where they appear as blind sacs. Their number is variable in different species. Pyloric ceca can be present as isolated structures for example in tuna (*Thunnus thynnus*), or as a unique structure as the pyloric gland of the sturgeon (*Acipenser transmontanus*) [48,56]. In general, pyloric ceca are storage organs allowing fish to eat infrequently avoiding starvation when feed is scarce and taking advantage of when feed resources are abundantly available [57]. 

Although fish have tongues, they are not nearly as well developed and capable of manipulating food items as the tongues of other vertebrates [57]. The tongue of the African bony-tongue appears roughly triangular in shape, and three areas can be distinguished as an apex, a body, and a root as in other fish species. The fish present a boney tongue like other osteoglossomorphs. This helps fish to expand their food source [58]. According to a functional point of view, we believe that the bone substrate in the tongue gives mechanical support in food intake, prehension, and deglutition or swallowing, and the underlying cartilaginous nuclei could represent useful cushioning elements to repair from pressure forces. The star or fused-shaped mesenchymal cells present within the primitive medullary cavities play a central role in bone remodeling. Finally, the presence of striated muscle fibers suggests an active role of the tongue in swallowing.

Alongside the tongue, the tubular organs show invaginations richly covered by taste buds. These invaginations represent pockets in which food particles are retained, increasing the ability to sense tastes. All these characteristics agree with the perception of taste function of the tubular organs and show a marked ability to select the type of food (chemosensory organs). 

The gastrointestinal system of African bony-tongue, taken as a whole, has the same histological features of a typical hollow organ consisting of the four main tunicae: mucosa, submucosa, muscularis externa, and adventitia or serosa. The folds of the mucosa are important for esophageal wall integrity from dilatation [52,55]. In the mucosa, the high number of muciparous caliciform cells represents an epithelial specialization to promote swallowing with lubricating and protective functions [47]. The presence of taste buds in the esophagus indicates a role in taste perception. The striated muscular fibers in the muscular tunica indicate that swallowing is a voluntary movement and continues with involuntary peristalsis. The muscular tunica is made up only of the external circular layer inducing the progression of the ingested food along the canal with the peristaltic movements.

Both *A. gigas* and *H. niloticus* have no valve between the esophagus and the stomach [52,59]. Not all fish species present a stomach; for instance, some members belonging to the Cyprinidae family are defined as “stomach-less” [36]. In some species, there is a thicking of the stomach wall. On the contrary, the African bony-tongue has a stomach made up of a proventriculus and a ventriculus (gizzard). Before its curved part, the proventriculus shows an oval section with a typical histological characteristic of hollow organs consisting of four main tunicae: the mucosa, the submucosa, the muscular layer, and the serosa. In the submucosa, the dense connective tissue sends both trophic and mechanical supporting septa to the glandular bodies of the overlying mucosa. The stomach muscular part (gizzard) appears dilated for feed receiving from the proventriculus. Here, digestion begins under the action of the secretion of digestive enzymes. The gizzard gets its common name from its muscular, gizzard-like stomach. It is a great modification of the stomach wall thickening, involved in digesting coarse foods into smaller pieces making them more assimilable. In the gizzard with a thicker muscular layer, the presence of the cuticula has a protective function on the underlying mucosa. It preserves digestive enzymes secreted in the proventriculus and from the food particles from mechanical erosion in accordance with what has been reported [60] in birds [61] and reptiles [62]. The three-dimensional network of connective tissue septa infiltrating the bundles of muscle fibers, has the function of connecting the muscle fiber cells and transmitting the mechanical forces generated by them. In addition, the connective tissue also has a trophic function by conveying the blood vessels through the septa. Once digested, the feed bolus, is deposited in the skull-ventral blind sac and is pushed through the pyloric ostium in the pyloric caeca, and the in the duodenum. It is allowed by muscular contraction.

In the pyloric ceca, the simple columnar epithelium covering the mucosa folds has apical membrane specializations typical of an absorbent epithelium. The presence of intercalated goblet cells has a protective function. The anastomosed villus-like folds in the mucosa form a labyrinthic structure in the pyloric appendix making a site of stasis of the food to promote the absorption of nutrients. Masses of lymphoid tissue in the mucosal lamina propria of pyloric ceca have been observed. These cells are particularly important in maintaining immune homeostasis within the intestine. According to our knowledge, the presence of these structures has been observed in this anatomical district of *H. niloticus*. for the first time. Probably, the related species *A. gigas* could also present these structures, but we did not find evidence in scientific literature. Moreover, the presence of well-organized lymphoid structures has never been documented in other fish species. On the other hand, the presence of scattered immune cells was found in the pyloric ceca of rainbow trout (*Oncorhynchus mykiss*) after oral vaccination [49]. Instead, what we observed in *H. niloticus* is similar to what has been found in the avian gut, where GALT (gut-associated lymphoid tissue) is present [50]. 

African bony-tongue intestine length is higher than stomach and pyloric caeca length differing from *O. bicirrhosum*, a fish belonging to the Osteoglossidae family, whose intestine has a similar length to the stomach and pyloric ceca [63]. These differences reflect the alimentary regime: the intestine is shorter in carnivorous [51] and longer in omnivores and herbivorous [36,52]. 

Variations in the structure of the different intestinal mucosa parts reflect the different intestinal parts’ functions, from the intermediate and posterior intestine, until the anus. For instance, the rectum mucosa appears covered by a simple columnar epithelium with abundant goblet cells compared to the rest of the intestinal segments. This helps the elimination of waste substances. In the rectum submucosa, the presence of secondary lymphoid organs suggests their importance as the first line of immune defense against ingested pathogens [53]. 

Regarding the gastrointestinal tract-associated glands, we analyzed the pancreas and the liver. In most fish species, the pancreas is usually diffused along the mesentery ligament [55,57,64]. In the African bony-tongue, it has a compact aspect. For this reason, it is possible to identify an endocrine and an exocrine part [58,59,60,65]. It is intriguing to notice that the pancreas is a distinct gland from the liver because other fish such as *A. gigas*, belonging to the same family of African bony-tongue, present an exocrine pancreas diffuse in the parenchyma of the liver [55]. The insular endocrine component we observed near the vessels has a structure completely comparable to the pancreas of mammals. 

Fish liver is a gland variable in form and size. Interspecies differences in liver lobe numbers have been found according to the different types of diet (herbivore, omnivore, carnivorous) [37,61,62,63,66]. African bony-tongue liver is bilobated as in siluriformes [67]. At a microscopic level, the liver of the African bony-tongue shows no evident interlobular septa for which it lacks a lobulation, as other members of the Arapaiminae family [55]. Finally, the presence of scattered melano-macrophage centers has been found as in other fish species [68,69]. These centers are formed by macrophage aggregates containing melanin, lipofuscin, and hemosiderin. They are involved in non-specific immune defense in the teleost fish [70]. 

## 5. Conclusions

In conclusion, the results of the present study give contribute to scientific literature regarding the morphology of the alimentary tract of the African bony-tongue. This study could be a good starting point for future investigations concerning physiological and immunohistochemical aspects. Expanding this kind of knowledge could be useful for researchers aiming to optimize husbandry, by providing a basis for developing optimized feeding protocols, nutritional requirements, and the digestive capacity of adult African bony-tongue, to further improve the growth performance of this species and ensure its conservation. Further studies are needed to understand the best way to feed the African bony-tongue in captivity, for instance, if food presentation (cropped or whole) could impact the food intake and behavior or the transit time inside the digestive tract.

## Data Availability

All data presented this study are available from the corresponding author, upon responsible request.
